# Oxide of Zinc in Diarrhœa

**Published:** 1874-04

**Authors:** William Berry


Oxide of Zinc in Diarrhœa.—*By William Berry, M.R.C.S.*—Oxide of zinc having been recommended by Dr. Brakenridge of Edinburgh, and Dr. Mackey of Birmingham in the treatment of infantile diarrhœa, as may be seen in the *Medical Times and Gazette* of February 15, and the *British Medical Journal* of July 12, I resolved to give it a fair trial, not only in children, but also in the autumnal diarrhœa of adults. So far I have every reason to be satisfied with it as a remedy for diarrhœa in children, especially in those in which the cause appears to be some irritation of the nerve centers presiding over the alimentary canal. In adults I have found it useful in cases of lienteric diarrhœa, but not so beneficial as the aromatic chalk powder of the pharmacopœia in ordinary cases.

| | | |
|---|---|---|
| ℞.—Zinci Oxidi | . . . . . . | gr: xxxii. |
| Mucilaginis | . . . . . . | |
| Syrup: Simpl: | . . . . . | aa. ʒ ij. |
| Aquæ ad | . . . . . . | ℥ ij. |

M. S. ʒ j. 3 tis horis.

